# Quality Controls in Ligand Binding Assays: Recommendations and Best Practices for Preparation, Qualification, Maintenance of Lot to Lot Consistency, and Prevention of Assay Drift

**DOI:** 10.1208/s12248-019-0354-6

**Published:** 2019-07-11

**Authors:** Mitra Azadeh, Perceval Sondag, Ying Wang, Maribeth Raines, Jeffrey Sailstad

**Affiliations:** 1 Bioanalytical and Biomarker Development, Shire, Lexington, Massachusetts USA; 2 Pharmalex Inc, Flemington, New Jersey USA; 3 BioMedicine Design, Pfizer, Andover, Massachusetts USA; 4 Laboratory Services at Pacific Biomarkers, Inc., Seattle, Washington USA; 5 Sailstad & Associates, Inc., Durham, North Carolina USA

**Keywords:** LBA, life cycle management, qualification, quality control, trending

## Abstract

Quality controls (QCs) are the primary indices of assay performance and an important tool in assay lifecycle management. Inclusion of QCs in the testing process allows for the detection of system errors and ongoing assessment of the reliability of the assay. Changes in the performance of QCs are indicative of changes in the assay behavior caused by unintended alterations to reagents or to the operating conditions. The focus of this publication is management of QC life cycle. A consensus view of the ligand binding assay (LBA) community on the best practices for factors that are critical to QC life cycle management including QC preparation, qualification, and trending is presented here.

**Electronic supplementary material:**

The online version of this article (10.1208/s12248-019-0354-6) contains supplementary material, which is available to authorized users.

## INTRODUCTION

The performance of a ligand binding assay is manifested in the performance of its quality controls during pre-study method validation and in-study sample analysis. Successful management of QC life cycle requires rigorous and established methodologies for preparation, qualification as well as for monitoring QC performance. Although the procedures and acceptance criteria for the preparation and qualification of the LBA QCs have been addressed in regulatory agency guidance documents and in LBA literature (1–3), such publications discuss these parameters to a limited extent to cover method development and pre-study validation phases. The subject matter of QC life cycle management and the factors critical to this process have not been previously addressed. Many questions regarding production and qualification of replacement batches of QCs in such a manner that lot to lot consistency is maintained and assay drift is prevented, remain unanswered. Additionally, the majority of bioanalytical laboratories lack established methodologies or statistical tools to trend QCs, a practice that is of paramount importance to monitoring performance and managing the life cycle of quality controls.

This publication aims to fill in the gap by providing guidelines for the management of LBA QC life cycle and its components including recommendations and best practices for QC preparation, qualification, and performance trending. A collective view of the LBA community on the subject matter is presented here. The authors’ goal is to help bioanalytical laboratories with defining QC qualification and lot to lot consistency guidelines in their standard operating procedures (SOPs). Assay- and study-specific requirements and specifications should be considered and detailed in the validated method or in the sample analysis plan of individual laboratory. This article primarily focuses on quantitative and qualitative LBAs such as pharmacokinetic (PK) and anti-drug antibody (ADA) assays although many discussions presented here are equally relevant to biomarker assays. All other assay categories are outside the scope of this publication.

## PREPARATION OF QUALITY CONTROLS

Reference standard, matrix pool, QC composition, equipment, and the production strategy are all critical factors in the preparation of LBA QCs; these are discussed in the sections below.

### Composition of Quality Controls

QCs should be prepared using a matrix that is similar and as close as possible to the study matrix. For example, if study samples are serum which has not been filtered, centrifuged, or charcoal-stripped, the QC matrix pool should also be unfiltered, non-centrifuged, and non-charcoal-stripped serum of the same species. Undiluted (100%) matrix should be used in the preparation of QCs so that QCs could be subjected to the same dilution steps [such as minimum required dilution (MRD)] as the study samples. Exceptions to this rule, such as substitution of a surrogate for a rare study matrix, require justification and are permissible only where matrix conservation is necessary. Examples of rare matrix include cerebrospinal fluid, synovial fluid, or ocular matrices from certain species which are often difficult to obtain in sufficient quantities. A recommended approach to mitigate matrix volume issue would be to prepare 2 of the 3 QC levels in a surrogate matrix and only one level in the study matrix. If a surrogate matrix is used, its equivalence to the study matrix should be demonstrated (4). It is preferable to use the same lot of the matrix as used for the preparation of calibrators, to generate QCs to enhance consistency and reproducibility if selectivity has been demonstrated during pre-study validation. The intermediate stock, which is the spiking solution of the analyte used to generate QCs, may be prepared in a diluent other than the study matrix such as water, buffer, or organic media so long as the final composition of the QC is at least 95% (*v*/*v*) matrix when water or buffer intermediate stocks are used and a minimum of 99% (*v*/*v*) matrix when organic solvents (e.g., DMSO or acetic acid) are used.

### Independent Preparation of QCs

Preparation of QCs should be independent of calibrators to prevent systemic spiking errors. In that regards, it is recommended that separate intermediate stocks and dilution steps be used to prepare QCs *versus* calibrators. It is also recommended that QCs be spiked independently at each level instead of through serial dilutions of the high QC. This is particularly important if calibrators are prepared via serial dilution of the high standard. Serial dilution of both the QCs and calibrators can mask dilutional linearity issues and should be avoided.Reference Standard

### Reference Standard

Reference standard in PK assays or the positive antibody control stock in the ADA assays must be within expiration at the time of QC preparation. Stability and expiration of the quality controls are independent of the reference material from which they are prepared and should be established separately because QCs are in a matrix different from that of the stock reference standard (5). Intermediate stocks which are diluted solutions of the reference standard in either the study matrix or a suitable diluent may be prepared, aliquoted, and stored for use in future QC or calibrator production. In such cases, the stability of the intermediate stock covering its storage window, that is from its date of preparation through the date of use, should be established. This may be done by comparing calibrators and/or QCs made from the frozen intermediate stock with those prepared using an original reference standard stock bottle.

### Qualified Matrix Pool

Proper selection of the matrix used in the preparation of QCs is critical to the quality of the assay and to the prevention of assay drift. This selection is particularly important in the ADA assays where the qualified matrix pool (QMP) is used for negative control (NC) which directly influences the plate-specific cut point. Appropriate screening and selection processes as well as the qualification criteria must be established and clearly defined in the validated test method or in an appropriate SOP. Recommendations for matrix qualification are summarized in the section below.

i. Qualification of the First Matrix Pool
  The first QMP is often generated as part of method development and formally qualified during method validation.

- Qualify adequate volumes of the QMP to last through multiple studies and phases. At a minimum, sufficient quantities of the matrix should be qualified to support pre-study validation and one or more bioanalytical studies.
- Store the QMP under the anticipated study sample storage conditions (e.g., − 20°C or − 80°C temperature).
- As the first step to matrix pool preparation, screen individual matrix samples or individual matrix pools (mixture of several individuals) by examining the signal generated by unfortified (unspiked) as well as analyte-fortified (spiked) individual samples.
- Examine the response from unfortified samples for background. Samples with abnormally high background (e.g., above the PK assay lower limit of quantitation (LLOQ) or above the ADA assay estimated cut point) should be excluded from the pool. For ADA assays, an abnormally low background may also be problematic.
- For quantitative PK assays, evaluate the spiked matrix samples for acceptance as defined in the test method or the laboratory SOP; relative error (RE) within ± 20% for acceptance of spiked matrix samples is recommended. It is recommended that individual matrix samples are spiked at a level between LLOQ and low QC (LQC) in a minimum of one run.
- For ADA assays, the importance of the blank signal (NC) must be emphasized. When no comparator lots are available as is the case with the first lot, it is recommended that individual matrix samples are evaluated in a Tier 1 (screen) assay and their raw responses assessed through comparison with the responses within their panel. All individual matrix samples with abnormally high or low response should be excluded from the replacement pool. Wherever possible, the matrix pool should also be compared against a panel of disease state matrix samples for its suitability.
- Example acceptance criteria for the pool background for a quantitative PK assay may be matrix response 2–3 folds lower than that of the estimated LLOQ. Example criteria for ADA assays are less than the estimated cut point or within a specific response range.

ii. Qualification of Replacement Matrix Lots
  Qualify a replacement lot of matrix using the same method used to qualify the first lot.
    Ensure the responses for the unfortified existing and replacement QMPs are comparable.
    For PK assays, replacement matrix lot should be compared with an existing qualified lot. This can be performed by spiking the reference standard at a level between LLOQ and LQC of the assay in a minimum of one run at *n* = 3. Analytical recovery (AR) of the reference standard in both existing and replacement lots of matrix should be within 80 to 120%. It is recommended that the difference between the measured concentrations of the reference standard in the two matrix lots does not exceed 10%.
    To qualify an ADA assay replacement QMP, individual matrix samples should be screened against the plate-specific cut point using a previously established cut point factor. All individual samples which are deemed as positive in the screen assay should be excluded from the replacement QMP. An alternative approach may be direct examination of the signal-to-noise (S/N) of each individual matrix sample; the matrix samples which are above the validated cut point factor should be excluded from the replacement QMP. The following are the recommended acceptance criteria for the replacement QMP: replacement matrix lot response should be within ± 10% of the existing matrix lot response, and if not, an assessment should be performed where a panel of individual matrix samples are compared for positive/negative screen outcome against two plate-specific cut points. One cut point is calculated with NCs from the existing QMP and the other, with NCs prepared with the replacement QMP. The screen test results (negative/positive status) using the existing *vs*. replacement plate-specific cut points should be comparable with the understanding that borderline samples may change Tier 1 status based upon the QMP response.
iii. Matrix Background
  Matrix background should be kept to a minimum in PK assays as it affects the assay sensitivity and may limit the quantitative assay range. For ADA assays, it is recommended that both the upper and lower limits of the matrix response range be established as soon as validation data are available.

- In non-competitive quantitative (e.g., PK) immunoassays, the matrix background should not exceed 1/3 of the LLOQ.
- In competitive immunoassays, the background should at least be 1.11 times the lowest calibrator. This is based on the *B*/*B*_0_ (lowest calibrator signal/zero calibrator) recommendation of 90% (100/90).
- In ADA and other non-quantitative assays, the general recommendations for matrix background are relative luminescence units (RLUs) ≤ 200 and absorbance ≤ 0.200. Some assays inherently have higher matrix background than those recommended above.

The lower limits of the matrix background are governed by the instrument response which may vary from instrument to instrument and from one laboratory to another.

### Equipment

- The equipment used in the preparation of QCs including pipettes should be verified for precision and should be within calibration.
- The equipment calibration process and frequency should be defined in an appropriate SOP.
- In some laboratories, an additional calibration check besides the scheduled periodic calibration is required immediately before the use of pipettes for the preparation of QCs; whereas, in other laboratories, a fresh calibration check is not required if the periodic calibration is still in effect.

## QUALIFICATION OF QCs

### General Run Acceptance Requirements

The general run acceptance criteria in this section apply to the qualification of both existing and replacement QC lots. Here, the terms baseline, legacy, or comparator QC lots are used interchangeably.

For quantitative PK assays:

1. QCs may be qualified against previously qualified frozen calibrators with established stability. If qualified frozen calibrators are not available, QCs should be evaluated against a freshly prepared calibration curve. In the latter approach, inclusion of a previously qualified set of QCs in the run serves to qualify the fresh calibration curve.
2. Qualification is performed through assessment of inter- and intra-assay precision (in all methods) and accuracy (in quantitative methods).
3. For quantitative and PK assays, QCs must meet the precision and accuracy criteria of coefficient of variation (CV) ≤ 20% and RE within ± 20% [or CV ≤ 25% and RE within ± 25% at LLOQ and upper limit of quantitation (ULOQ)].

For ADA and other qualitative assays:

1. QCs should meet the CV criterion of ≤ 20%.
2. The response for both existing and replacement QC should be within the established response range for that control level. If a replacement QC lot falls outside of its signal range, laboratories must conduct troubleshooting and determine the root cause. Potential sources of drift for ADA positive control (PC) and NC signals include (a) spiking error, (b) poorly selected matrix pool, and (c) incorrectly established signal range. Subsequent troubleshooting should follow the order above: first prepare a new batch of QCs, and if repeat preparation fails, qualify a new lot of QMP and subsequently repeat the PC and NC preparation. If PC and NC are still outside their signal ranges, re-evaluate the established signal ranges by including additional data from in-study runs. Laboratories should establish the post-validation signal ranges appropriately and based on an adequate number of runs to avoid setting too narrow a range or setting a range that may prove unsuitable over time.
3. The criterion of high positive control (HPC) > low positive control (LPC) > cut point > NC should be met.

For all assays:

1. It is recommended that a minimum of 3 sets of each existing and replacement QCs be included in each qualification run. Individual laboratory procedures may vary in this requirement.
2. A minimum of 2/3rd of all qualification runs should have acceptable performance as specified above. A summary of these qualification requirements and recommendations is presented in Table I.
3. Both existing and replacement lots of QCs must meet the general run acceptance criteria stated above.
4. If a replacement lot of QCs fails the above-stated acceptance criteria while the comparator (existing) lot passes, the failed replacement lot should be discarded, and another batch prepared. When a single level of replacement QC fails, it is permissible to replace that level alone.
5. If the existing QC lot fails run acceptance criteria during qualification of a replacement lot, it should be reanalyzed to confirm results. If the existing lot fails again upon repeat analysis, it is unusable as a comparator. In such cases, the procedure and specifications stated in Qualification in the Absence of an Existing Qualified Lot section should be followed.

**Table I:** Comparison of Qualification Requirements for Replacement QC Lots

Qualification of	No. of runs	No. of days	No. of replacement and comparator QC sets per run	No. of analysts	No. of acceptable qualification runs	% difference between the two lots	Additional requirements
1st lot QCs	≥ 6	≥ 2	≥ 3	≥ 2	≥ 4 of 6	NA	Criteria stated under General Run Acceptance Requirements must be met
Replacement lot against baseline lot	≥ 2	≥ 2	≥ 3	≥ 1	2 of 2	≤ 10% (difference between existing and replacement lots) for quantitative assays Based on acceptance response range for non-quantitative methods	Minimum of 1 analyst in two 2 days or 2 analysts in 1 day Criteria stated under General Run Acceptance Requirements must be met
Replacement lot in the absence of a baseline lot	≥ 6	≥ 2	≥ 3	≥ 2	≥ 4 of 6	≤ 10% (difference between the two independently prepared lots) for quantitative assays Based on acceptance response range for non-quantitative methods	2 independent lots prepared by 2 analysts Criteria stated under General Run Acceptance Requirements must be met

### Qualification of the 1st Lot of QCs

The first lot of QCs, also referred to as the baseline or legacy lot, is typically qualified as part of accuracy and precision (A&P) assessment in pre-study method validation. A minimum of 6 independent runs with 3 independent sets of each QC level (3) over a minimum of 2 days, by a minimum of 2 analysts is recommended for the qualification of the pre-study method validation QC batch. A minimum of 4 out of 6 QC qualification runs should have acceptable performance to qualify this lot (see Table I). Where possible, it is also recommended that this lot is bridged to the method development QCs. The acceptance criteria for such bridging evaluation are to be established by individual laboratories; difference of ± 10% or better is recommended. The run requirements specified under General Run Acceptance Requirements must be met.

### Qualification of a Replacement Lot of QCs

i. Qualification Against an Existing Qualified Lot
  Beyond pre-study method validation, every time a replacement lot of QCs is prepared, it should be qualified against a previously qualified (existing) lot. It is critical that the existing and the replacement lots of QCs are evaluated against the same frozen or fresh calibration curve for reliable comparability assessment. A replacement lot of QC should be qualified in a minimum of 2 independent qualification runs. Qualification runs may be performed on the same day by multiple (minimum of 2) analysts or by the same analyst on multiple (minimum of 2) days. It is recommended that a minimum of 3 independent sets of the replacement QCs and a minimum of 3 sets of an existing lot of QCs are included in the same run. An independent set is defined as one prepared from an independent frozen QC aliquot. Use of a single QC aliquot for the preparation of three sets in the same run is discouraged during QC qualification. Individual laboratories may set criteria that are different from those recommended here such as 3 independent runs instead of 2 depending on their assessment of the variability and risk involved. Qualification of a replacement lot of QCs in quantitative LBAs should be based on the criteria stated under General Run Acceptance Requirements but also based on its comparability to an existing qualified lot. Example comparability criteria may include difference of ± 10% or better between existing and replacement lots. The % difference is determined using the measured concentrations of the existing and replacement QCs with the equation below.

$$ \frac{Concentration\ of\ Replacement\  Lot- Concentration\ of\ Existing\  Lot}{Mean\ Concentration\ of\ Existing\ and\ Replacement\ Lots}\times 100 $$

For ADA assays and other non-quantitative LBAs, the criteria stated under General Run Acceptance Requirements for ADA and qualitative assays should be met for both existing and replacement lots. Additionally, the responses of both existing and replacement lots of PCs and NCs should be within their respective established signal ranges.

ii. Qualification in the Absence of an Existing Qualified Lot
  There may be instances where a legacy or a previously qualified lot of QCs does not exist because such lot has been exhausted or has expired. In the absence of a comparator, it is recommended that two separate replacement batches of QCs be prepared independently from separate intermediate stocks, each by a different analyst. For practical purposes, one lot may be designated as primary and may be of a larger size, and the second lot may be of smaller scale prepared only to serve as comparator for the qualification of the primary lot. Comparative testing of the primary and secondary lots should be performed by 2 analysts each utilizing an independently prepared calibration curve, each performing 3 qualification runs over 2 days, for a total of 6 runs. A minimum of 4 out of 6 (2/3rd) QC qualification runs should have acceptable performance. General Run Acceptance Requirements stated above should also be met. The two replacement lots of QCs should meet the % difference criteria of ± 10 or better for either lot to be acceptable. A summary of these specifications is presented in Table I.

### Qualification of QCs Prepared in Matrices Containing Endogenous Analyte

In quantitative PK assays where the matrix contains an endogenous homolog of the analyte, the concentration associated with the matrix blank may be significant in which case it should be factored in. In the subtractive approach, the concentration associated with the matrix blank is deducted from the measured concentrations of QCs before QC values are reported. In the additive approach, the measured concentration of the matrix blank is added to the spike concentration of the QC to establish its adjusted nominal value. Marcelletti *et al.* (6) presented several case studies involving direct comparison of spiked recovery results using additive and subtractive approaches where recoveries of a number of biomarkers with appreciable endogenous target levels were evaluated. Based on these published case studies, subtraction is the preferred approach, and addition is discouraged. It is recommended that a minimum of 3 sets of matrix blank samples are included in each run and their mean measured concentration used for the adjusted value computations. It is recommended that QCs with endogenous analyte be qualified in a minimum of 30 runs over 10–20 days by a minimum of 2 analysts using ≥ 3 sets of QCs per run. Run acceptance criteria for the qualification of this category of QCs are the same as those stated under General Run Acceptance Requirements for quantitative PK assays.

### Qualification of QCs with Unknown Nominal Concentration

If the nominal concentration of the QC stock is unknown as in the case of unpurified proteins and some commercial control stocks, or when crude serum or plasma are used as the source of blood factors in hematology assays, the nominal concentration must be established based on the observed mean values from multiple runs on multiple days performed by multiple analysts. The appropriate specifications for such determinations are assay-specific and must be established as part of pre-study method validation. QCs are to be prepared by spiking the stock into a pre-qualified matrix pool or an appropriate diluent at specified dilutions. The mean measured value for each QC from all passing runs would set the nominal concentration of that QC. It is recommended that a minimum of 30 acceptable runs, performed over 10–20 days by a minimum of 2 analysts are used to set the nominal concentration of the QCs. Each run is to include ≥ 3 sets of QCs. Following the initial determination, the recommended criteria for acceptance of QCs in daily assay runs as well as for the qualification of future replacement QC lots is ± 20% of established nominal value. The observed concentration of any replacement lot must be within this established range. Due to the inherent variability associated with serum- and plasma-derived factors, it may be necessary to assign a temporary nominal value to each QC level based on the initial 20 to 30 run data and subsequently re-evaluate the suitability of this nominal value as additional data become available. In case of assays with such inherent variability, the QC acceptance ranges may need to be re-assessed and re-established periodically. The General Run Acceptance Requirements for quantitative PK assays stated above must also be met.

### Qualification of QCs with Dissimilar Nominal *vs*. Measured Units

Enzymatic activity assays are amongst those in which the nominal and measured QC values have different units. In these assays, the nominal (spike) concentration of the enzyme drug is typically provided in ng/mL or μg/mL; whereas, the measured value of the spiked QC sample is in activity units of nmol/h/mL or nmol/h/μg. Such disparity in units poses a unique challenge in the qualification of QCs as it does not allow for the computation of accuracy. In these cases, the limitation may be overcome by establishing a nominal activity value for the QC. The nominal QC activity value for each level can be established based on the mean measured activity from a minimum of 30 acceptable runs, by a minimum of 2 analysts over a minimum of 10–20 days. A minimum of 3 sets of QCs should be included in each run. The mean measured activity from all passing runs would establish the nominal QC activity value and would allow for the computation of %RE for individual QCs using the equation below (7):

$$ \% RE=\frac{Measured\ Activity\ of\ Individual\  QC- Nominal\  QC\  Activity\ {Value}_{m30}\ }{Nominal\  QC\  Activity\ {Value}_{m30}\kern0.5em }\ x\ 100 $$

Where *Nominal QC Activity Value*_*m*30_ = *Mean measured activity based on* 30 *runs*

Following the initial determination, the criterion of nominal QC activity value ± 20% should be used for both daily run acceptance as well as for qualification of replacement QC lots. Table II below summarizes considerations for the special categories of QCs discussed above.

**Table II:** Summary of Requirements for the Specialty Categories of Quality Controls

Qualification of	No. of runs	No. of days	No. of QC sets per run	No. of analysts	Additional requirements
QCs in Matrices with Endogenous Analyte (e.g., PK assays for recombinant growth factor drugs or cytokine biomarker assays)	≥ 30	10–20	≥ 3	≥ 2	Inclusion of zero QC/blank matrix for correction of other QCs Implementation of subtractive method is recommended for quantitative determination Criteria stated under General Run Acceptance Requirements must be met
QCs with Unknown Nominal Concentration of Analyte Stock (e.g., blood factor assays)	≥ 30	10–20	≥ 3	≥ 2	Empirical determination of the QC concentration through repeat evaluation Criteria stated under General Run Acceptance Requirements must be met
QCs with Dissimilar Nominal *vs*. Measured Units (e.g., enzymatic activity assays)	≥ 30	10–20	≥ 3	≥ 2	Establishment of the nominal QC activity value based on the mean of observed measurements to harmonize nominal v. measured units Criteria stated under General Run Acceptance Requirements must be met

## REGULATORY PERSPECTIVE ON QUALITY CONTROLS

Quality controls for LBAs should be prepared by fortifying a qualified matrix pool with a known concentration of the reference standard (for PK assays) or dilution or concentration of the positive control antibody stock (for ADA assays). The general guidelines for the LBA quality control composition, target values, and acceptance criteria have been outlined in United States Food and Drug Administration (FDA), European Medicines Agency (EMA), the Japanese Ministry of Health, Labor and Welfare (MHLW), and the Brazilian Sanitary Surveillance Agency (ANVISA) guidance documents (3,8–11). Table III summarizes the agency requirements for quantitative (PK) assays with respect to preparation and composition of assay quality controls:

- QCs should be prepared in accordance with the established method.
- For quantitative LBAs such as PK assays, a minimum of three QC levels at the low, medium, and high levels are required. The high QC (HQC) should be prepared at approximately 75% of the ULOQ, the medium QC (MQC) should be spiked at a level equivalent to the geometric center of the quantitative range (midpoint of the LLOQ and ULOQ positions), and the LQC should be spiked at three times the LLOQ or lower. A QC should always be bracketed by calibrators at the upper and lower ends, or it could not be accepted even if it meets the %CV and %RE acceptance criteria.
- For qualitative LBAs such as ADA assays, high and low positive controls (HPC and LPC) are required, and a medium control is generally included in pre-study validation but optional for in-study runs (12). No specifics are provided in the agency guidance for the HPC spike level. The guidance recommendation for LPC is that it be prepared at a level such that it has an approximately 1% failure rate (12). This means one out of every 100 LPCs is expected to fall below the screening plate cut point.
- All QCs should be stored as single use aliquots under the conditions anticipated for study samples and in accordance with the validated test method.

**Table III:** Comparison of Regulatory Agency Requirements for Quantitative Assay Quality Controls

Parameter	Specifications			
	FDA (USA) (2018 Guidance)	EMA (Europe) (2012 Guidance)	MHLW (Japan) (2014 Guidance)	ANVISA (Brazil) (2003 Guidance & 2012 RDC #27 Resolution
QC levels–PK assay	3	3	3	3
Replicates/QC level	2	2	2	2
Inclusion of LLOQ and ULOQ level QCs in method validation	Required	Required	Required	Required
QCs and calibrators prepared from independent stocks	Required	Required	Not addressed	Not addressed
Minimum number of runs/days to validate A&P QCs	6 runs ≥ 1 day	6 runs	No specifications provided	≥ 3 runs
RE	± 20% (± 25% for LLOQ and ULOQ only)	± 20% (± 25% for LLOQ and ULOQ)	± 20% (± 25% for LLOQ and ULOQ)	± 15% (± 20% for LLOQ and ULOQ)
Total error	± 30% (± 40% for LLOQ and ULOQ)	± 30% (± 40% for LLOQ and ULOQ)	± 30% (± 40% for LLOQ and ULOQ)	Not addressed
CV	CVs < 20% (25% for LLOQ and ULOQ)	CVs < 20% (25% for LLOQ and ULOQ)	CVs < 20% (25% for LLOQ and ULOQ)	CVs < 20%
Specifications for LQC/LLOQ or LQC/blank ratio	LQC = 3X LLOQ	LQC < 3X LLOQ	Addressed but no specificiations provided	Specified: LLOQ ≥ 5 times blank sample
Trending QCs and monitoring drift	Recommended	Not addressed	Not addressed	Not addressed

Additional recommendations which are not agency requirement but equally good practices include the following:

- Preparation of QCs in sufficient quantities to support method validation, short-term and long-term stability studies, and at least one bioanalytical study.
- Preparation of QCs in larger quantities to span several years and a multitude of studies provided that stability evaluation to cover the storage window has been conducted or is in progress and will be available prior to reporting the study sample results.

## QC PERFORMANCE TRENDING

The Manufacturing Process Validation guidance released by Food and Drug Administration in 2011 (13) introduces the concept of lifecycle management for pharmaceutical processes and recommends monitoring the quality of each process as soon as its performance specifications have been established and validated. Sondag *et al.* and Schofield (14,15) have also recommended the incorporation of lifecycle management for assays. Laboratories are responsible for developing internal guidelines for assay trending. Such guidelines should be defined *a priori* to prevent in-study issues; statistical process control (SPC) is essential to achieving this goal. Combining graphical and statistical tools in QC trending is recommended. Trending should ideally start as early as pre-study validation, or otherwise no later than with the first bioanalytical study. The 2018 FDA Bioanalytical guidance has addressed the need for monitoring the performance of QCs as well as for evaluating the underlying causes of any drift, although no monitoring guidelines have been provided by the agency. It should be noted that CLIA-specific trending recommendations have been provided by the agency, and to an extent, they are also applicable to LBAs as CLIA also calls for multiple runs with multiple QC sets over multiple days and by multiple analysts. QC trending may be real-time or indirect; Scherder and Giacoletti (16) have discussed the difference between the two approaches. Most SPC tools are designed to detect shifts early enough so that appropriate corrective actions may be devised and implemented.

### Statistical Process Control

Statistical Process Control is a QC trending methodology for ensuring that a system continuously operates as intended. The two main objectives of SPC analysis are (a) verification that the process is in a state of statistical control, and (b) measuring its capability to produce results that fall within certain specifications.

The following steps are recommended for SPC:

1. Use the initial QC data (e.g., *n* = 30) to calculate the mean and the standard deviation (SD)
2. Establish QC limits using the mean and the SD
3. Establish all other QC rules
4. Monitor the process for trends using a QC chart and the established QC rules

#### State of Statistical Control—QC Charts

A process or a method is under a state of statistical control if it allows for the prediction of future results. This means that every source of variability in the process must be understood. QC charts are used to monitor unexpected variations and shifts (appearance of a systematic bias) in the process. Control charts are helpful in identifying unwanted assay events provided that anomalous results could be correlated with alterations to assay parameters (a different diluent lot, matrix lot, or analyst.)

The main types of control charts used in QC trending are run charts, Xbar-R charts, and individual control charts of the group mean (run charts on mean).

**Fig. 1:**
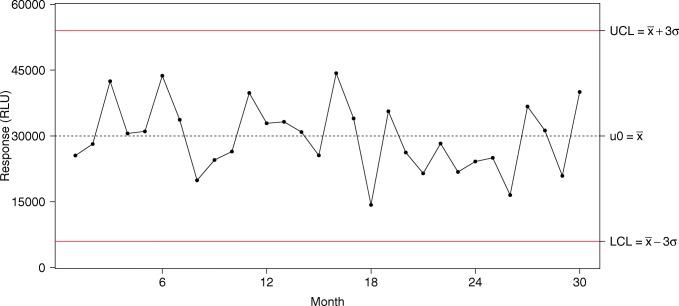
Example of a Levey-Jennings chart. The plain horizontal red lines are the lower (LCL) and upper (UCL) control limits; the dashed horizontal black line is the observed mean (μ_0_)

**Fig. 2:**
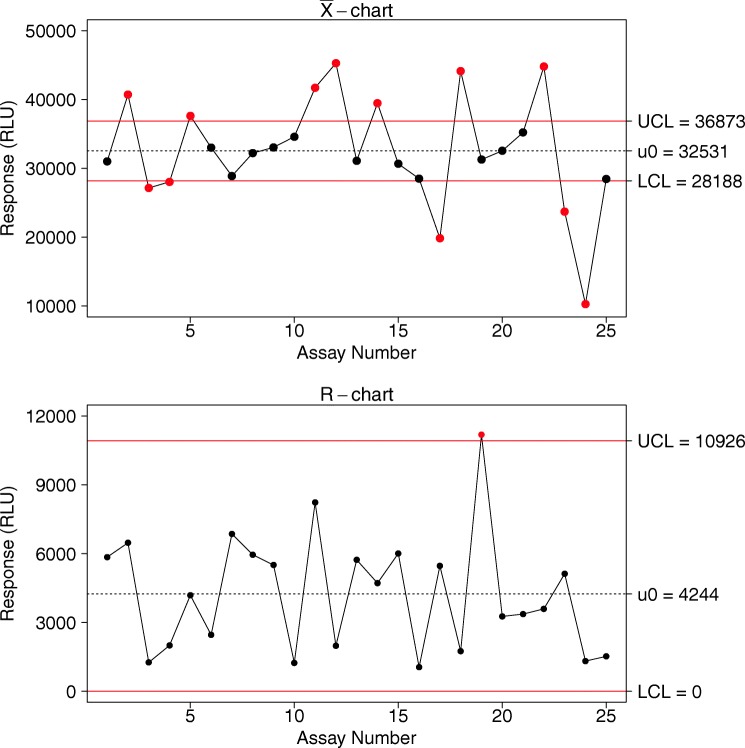
Example of a Xbar-R chart. The top panel is the Xbar chart, presenting the mean of each assay; the bottom is the R chart, presenting the observed range within each assay. For both charts, the plain horizontal red lines are the lower and upper control limits; the dashed horizontal black line is the observed mean across assays

Run charts are the simplest in which every trending measurement is plotted. A run chart is usually combined with a moving-range chart that presents the difference from one measurement to the next. Levey-Jennings (17) is the most commonly used run chart for LBAs (Fig. 1). Another common type of control chart is the Xbar-R chart. This chart type presents the trend in terms of average and range of each group. The control limits for such charts are built based on intra-assay variability, making them less suitable for LBA QC trending. Figure 2 is an example of Xbar-R chart. This figure is also a demonstration of how these chart types may lead to false alarms. An alternative to Xbar-R chart would be an individual control chart where the plotted values are the average of each assay; these are run charts on mean. In contrast to the Xbar-R charts, control limits for run charts on mean are calculated based on the inter-assay variability. It is still useful to add an Xbar-R chart to the run chart on mean to monitor the intra-assay variability trend.

Classically, the control limits are calculated as $\overline{x}\pm 3\sigma$ where $\overline{x}$ is the average of the observed data and σ (sigma) is the standard deviation of the population. When *σ* is unknown, SPC software will, by default, estimate sigma by $\overline{MR}/\left(2/\surd \left(\pi \right)\right),$where $\overline{MR}$ is the average observed moving range and 2/ √ (*π*) is the expected value for the range between two values from a standard Normal Distribution. $\overline{MR}/\left(2/\surd \left(\pi \right)\right)$is a representation of the short-term variability. For Levey-Jennings charts, sigma is an estimate of the long-term variability of the assay (Fig. 1).

Computation of ± 3 sigma at early stages may be challenging since it is a poor representation of the true long-term variability. Alternatively, Bayesian statistics may be applied to allow the calculation of the predictive distribution of quality control samples in method validation and beyond. Statistical experts may be consulted in the early stages of trending to establish preliminary control limits. Once sufficient data are available to accurately estimate sigma (e.g., at least 30 measurements over at least 10 to 12 months), advanced methodologies may no longer be necessary, and ± 3 sigma limits could be applied. Control limits should then be re-assessed regularly until a higher number of data points (e.g., 90) is available. Control limits may then remain fixed for longer periods of times; for example, for 12 or 24 months until a change is introduced in the process and re-assessment becomes necessary.

**Fig. 3:**
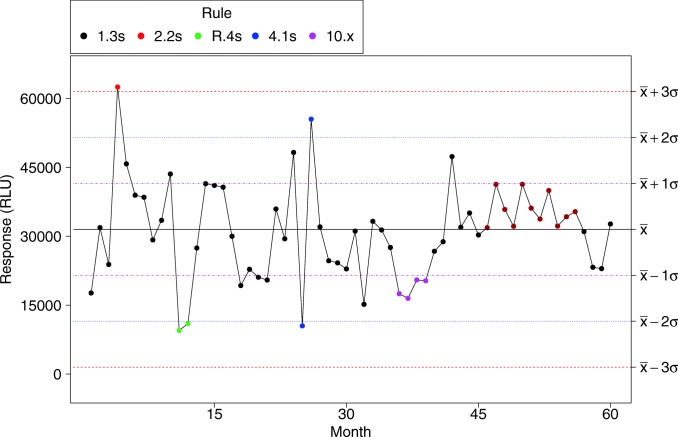
Westgard detection rules presented all in one plot. The plain black line is the observed mean, pink dashed line is the ± 1 sigma zone, the blue dotted line is the ± 2 sigma zone, and the red dashed line is the ± 3 sigma zone. Each colored marker represents an alert according to the associated rule

In addition to the calculation of control limits, most SPC software such as JMP® and Minitab® allow for the application of Western-Electric (WE) rules to accelerate the detection of a process drift (18). These rules are useful but run the risk of higher false alarm rates. In combination with Levey-Jennings chart, Westgard rules are a modification of the Western-Electric rules that are better adapted to laboratory practices for their reliance on SD (19). The most common Westgard rules are detailed below but also demonstrated in Fig. 3:

- 1.3s: 1 run falls outside the +/− 3 sigma limits
- 2.2s: 2 consecutive runs fall outside the +/− 2 sigma limits on the same side on the mean
- R.4s: 2 consecutive runs fall outside the +/− 2 sigma limits on alternate side on the mean
- 4.1s: 4 consecutive runs fall outside the +/− 1 sigma limits on the same side on the mean
- 10.x: 10 consecutive runs fall on the same side of the mean

As in WE, Westgard rules tend to increase the rate of false alarm and undue investigation. Laboratories should use these rules at their discretion and based on the performance of the assay. These rules provide additional monitoring tools and are not regarded as hard statistical rules (20). The alarms generated by these rules do not always require a full investigation (see Fig. 3). In this regard, SPC tools are for early detection of shifts so that corrective actions could be implemented in a timely manner.

#### Capability Assessment

Another important feature of SPC is that it is a measure of the capability of the laboratory for meeting specifications. The most common way to measure capability is the Process Capability Index (Cpk), calculated by:

$$ Cpk=\min \left[\frac{\  USL-\overline{x}}{3\hat{\sigma}},\frac{\ \overline{x}- LSL}{3\hat{\sigma}}\right] $$

**Fig. 4:**
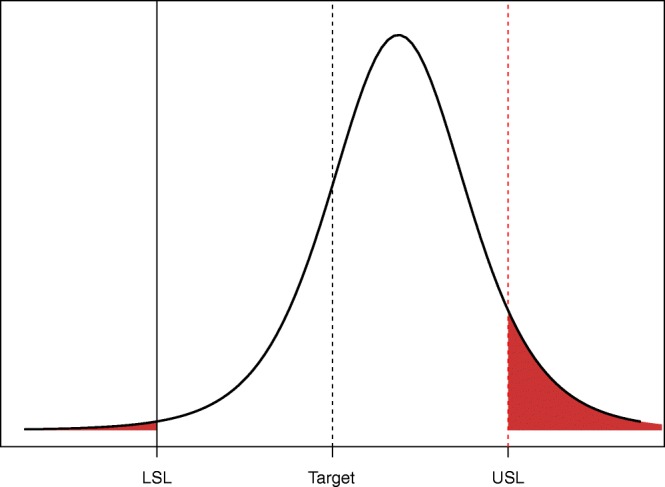
Predictive distribution of response. The dashed vertical line represents the target and the plain vertical lines represent specification limits. Red zones represent the probability that a future sample result would fall outside specification limits

Where LSL and USL are the lower and upper limit of specification, respectively, and $\hat{\sigma}$ is an estimate of the standard deviation (21). If Cpk is lower than 1, the control limits ± $3\hat{\sigma}$ would be wider than the specification limits and therefore, not applicable. A more direct way to estimate the probability that future results will remain within the acceptance range is to make a prediction based on the available data and the distribution of future QC values and to compare them with the specification limits to calculate the probability of success (PoS). Bayesian statistics allow for calculation of the predictive distribution of future QC results and thereby calculation of the PoS. This approach takes into account all sources of variability (14). With a well-balanced data set, this distribution can be obtained directly (22). Figure 4 presents an example of predictive distribution of response.

### Using Levey-Jennings Principles to Trend QC Performance in Ligand Binding Assays

LBA QCs can be trended using parameters relevant to the assay performance characteristics for example, departure from the accuracy acceptance range (%RE within ± 20%) in quantitative LBAs or departure from established response ranges for PCs and NC in ADA assays. For ADA assays, the response can be either the raw or normalized response. The following section provides examples of both ADA and quantitative PK assay QC trending.

#### Trending QC Performance for ADA Assays

**Fig. 5:**
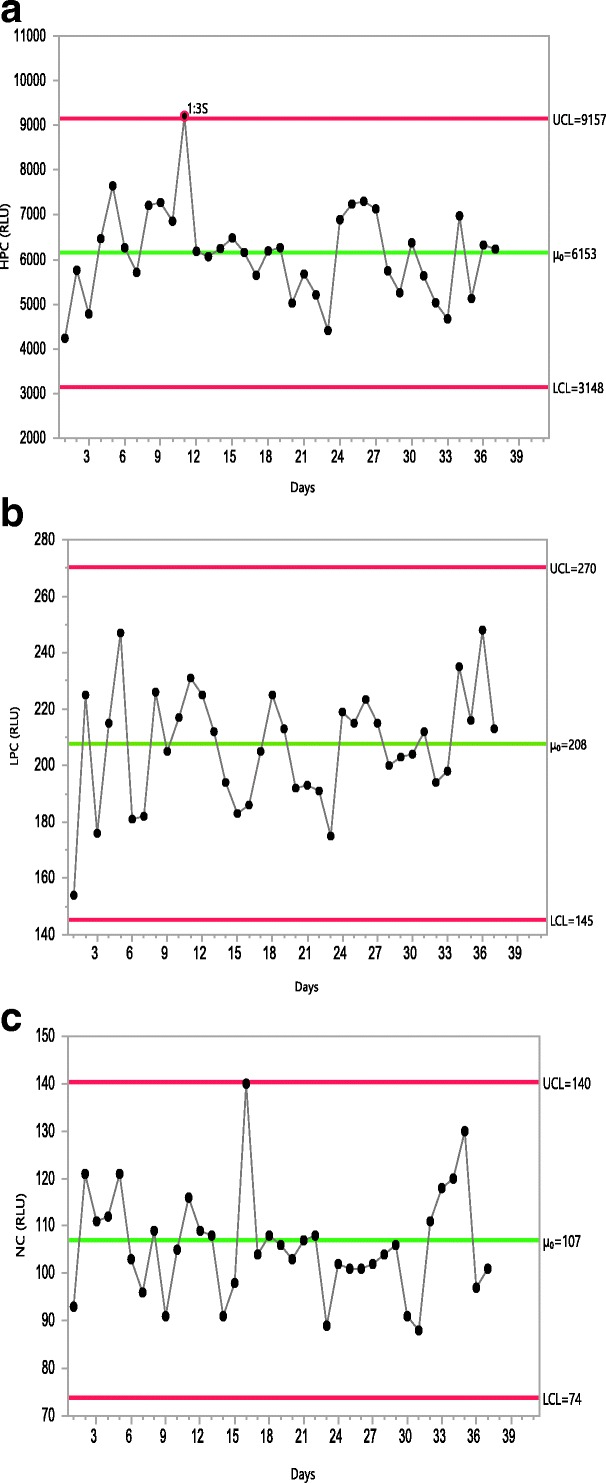
Example of trending of PCs and NC in ADA assays. In this simulated example, the PC and NC performances are trended using Levey-Jennings plots, with UCL and LCL of PCs established as observed mean response (μ_0_) ± 3 Sigma (while the upper limit of NC was established using mean response + 3 Sigma. In general, NC UCL is critical to restrain the overall background response levels of the assay, while LCL of LPC in some cases could overlap or be below the assay cut point, it is restrained by the assay cut point. Once pre-study method validation has been completed, the UCL and LCL limits are fixed for monitoring the assay performances during in-study sample analysis. In this plot, the controls which exceed their limits have been marked in red circles. For the NC, there is an upward trend in performance by day 16 and again by day 35; this trend was reversed in subsequent runs. Had such trend continued, it would have indicated a drift and would have warranted an investigation. These analyses were performed using JMP software. **a** HPC (3 Sigma). **b** LPC (3 Sigma). **c** NC (3 Sigma)

For ADA assays, the acceptable QC ranges are typically established during pre-study method validation and applied to in-study sample analysis. Ranges are calculated statistically using the cumulative mean QC values from pre-study validation runs (23) to define the upper control limit (UCL) and the lower control limit (LCL) of the positive controls as mean ± 3 sigma, and the upper limit of the NC as mean + 2.33 sigma. One might also consider limits that would afford a 1% failure rate for PCs. The 1% failure rate would not necessarily lead to the assay rejection considering that both PCs at a given level have to fail to result in run failure. ADA QC performance may be trended against these ranges. Figure 5 presents examples of ADA PC performance trending over time. Here, data from pre-study method validation were used to calculate UCL and LCL for PCs and UCL for the NC. Subsequently, these limits remain fixed for the life cycle of the assay.

#### Trending QC Performance for PK Assays

**Fig. 6:**
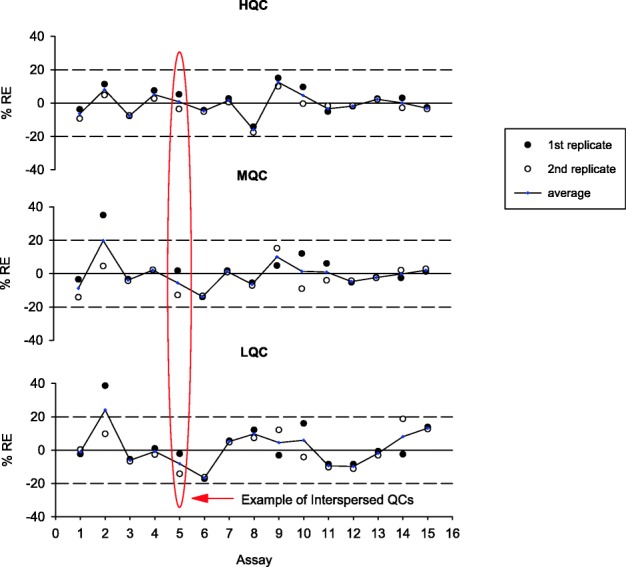
Example of intra- and inter-assay performance trending of three QC levels in a PK assay. Note that in this example, concentrations at each QC level in each assay run were evaluated using %RE against their nominal values. Since two sets of QCs at each level (n = 2) were included in every plate, plotting positional QCs allowed for their comparison and assessment for drift. At each QC level, the open and closed circles represent %RE for the two separate positional QCs on the plate. Red oval highlights the variability between interspersed positional QCs in Run 5

For PK assays, typically three levels of QC (HQC, MQC, and LQC) which span the quantitative range of the assay are included for monitoring run performances (1,24). The accuracy of QC data is assessed using %RE with acceptance limits of ± 20%. An example of PK assay QC performance trending is presented in Fig. 6. In this example, both intra- and inter-run performance are plotted and evaluated for drift (3).

#### Trending Performance and Establishing Control Limits for Biomarker Assays

As in PK assays, biomarker assays also aim to include three levels of QCs (HQC, MQC, and LQC) that span the quantitative range. These QCs could be used to monitor the assay performance. When endogenous QCs are used, it may not always be possible to construct three QC levels in which case, fewer QC levels are acceptable. In the absence of true reference standards, accuracy for biomarker assays should only be referred to as relative accuracy. Note that the contribution from the endogenous analyte may need to be factored in where applicable. Endogenous analyte enhances the nominal value, and unless tightly controlled, it may change between lots. Performance of biomarker QCs can be monitored in several ways:

- as %RE
- as measured value
- as Standard Deviation Index (SDI)

When the nominal QC values are known, the relative accuracy of the QC concentration is evaluated using %RE, and the trending limits are typically restrained within ± 20% as for the PK LBAs. In such instances, trending could be performed as shown in the PK assay example provided in Fig. 6.

**Fig. 7:**
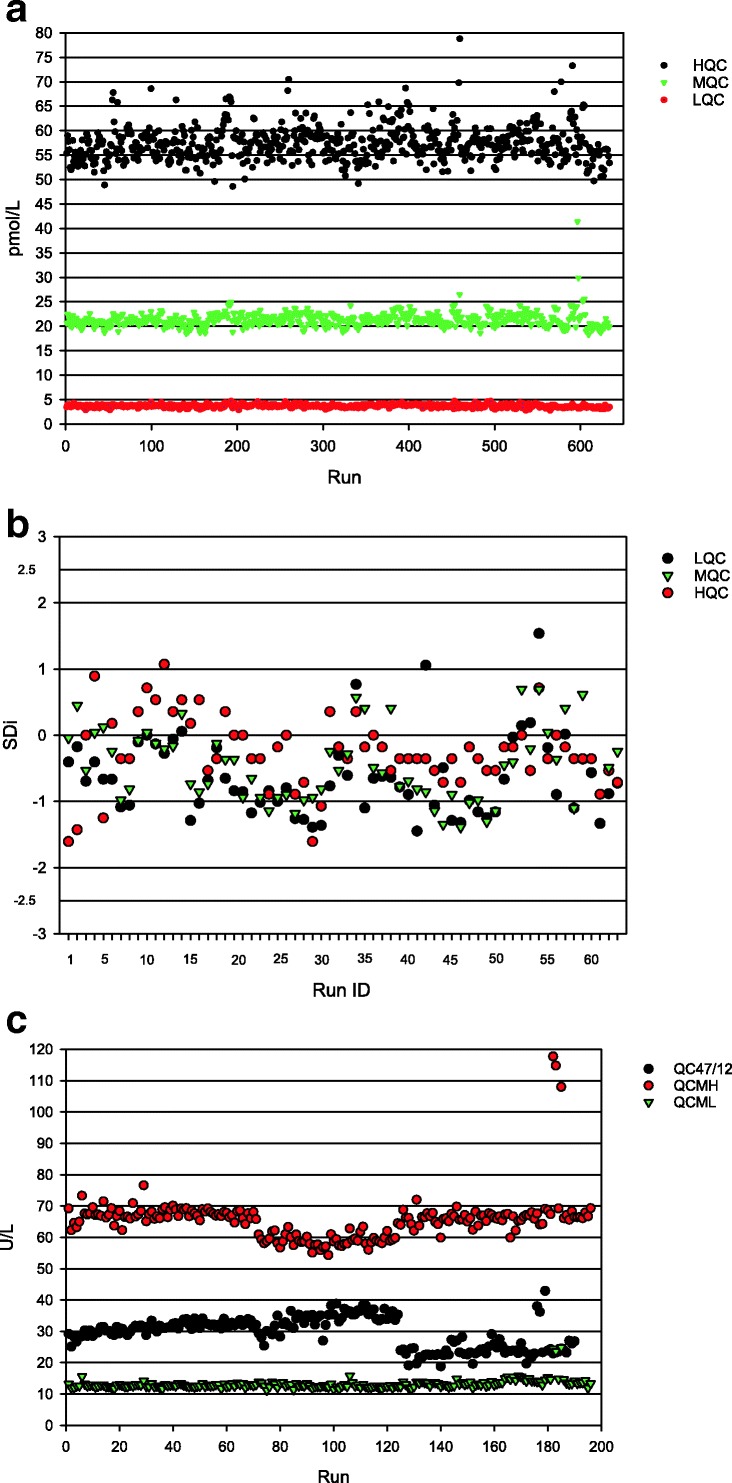
Laboratory examples of QC performance trending in biomarker assays. Laboratory QC monitoring data from two different biomarker assays, assay 1 (**a**, **b**) and assay 2 (**c**) analyzed over 987 and 686 days, respectively. **a** QC values of assay 1 plotted *versus* the analytical run number. Each plate has two sets of QCs per run and individual QC results are listed consecutively such that the total number of runs is half of what is represented. **b** A subset of the same data used in **a** but expressed as SDI values and listed relative to the Run ID. **c** Measured QC values of assay 2 plotted *versus* analytical run ID demonstrating shifts in performance due to reagent lot changes (at Run 72) and an assay performance issue (at Run 125). Note: in these examples, QCs from failed plates were included. The plots were performed in Microsoft Excel

Figure 7a presents laboratory data of a well-controlled biomarker assay in which measured values were used for trending. In this example, measured values are plotted against run IDs. This is a useful methodology which allows for the detection of shifts and the assessment of whether the shift is limited to one or multiple QC levels. Figure 7c shows a similar example of a measured value plot against run ID using laboratory data from an altogether different assay where two distinct shifts were observed in QCs. The first shift was observed with QCMH and QC47/12 at Run 72 where there was a change in the reagent lot, and the second, at Run 125 when yet another reagent lot change was implemented. In this latter case, QCMH (a commercial QC material) values returned to previous levels after Run 125, but QC47/12 (an in-house serum pool) values were lower than ever observed. The shift at Run 125 triggered an internal investigation which led to the identification of a manufacturing change in the solid phase coating antibody lot. A more rigorous plate washing program later corrected for this shift.

When the nominal QC values are unknown, the SDI is the recommended trending approach. The SDI is a value used in clinical laboratories to compare proficiencies between testing sites; it measures accuracy relative to the mean and precision of the assay. This index represents the number of standard deviations of each result from the QC mean. SDI is determined as follows:

$$ Standard\ Devation\ Index=\frac{{\overline{X}}_{in}-{\overline{X}}_{group}\ }{\ {\sigma}_{group}} $$

$$ {\overline{X}}_{in} $$
*Mean QC values from an individual run*
$$ {\overline{X}}_{group} $$
*Mean of ≥ 30 runs*
*σ*_*group*_: *Standard deviation of ≥ 30 runs*

When using SDI for assay trending, a preliminary mean and SD may be assigned using pre-study validation runs. These parameters may be further fine-tuned based on in-study values. The mean should be calculated from a statistically significant number of analytical runs (*n*) using a minimum of 30 runs over 10–20 days. This is a variation of the CLSI guidelines which recommend a 20 × 2 × 2 approach (20 days, 2 runs per day, 2 sets) for defining the precision of a method (25–27). Once the final SD has been established, it should remain constant for the life of the assay across future QC lots.

Figure 7b is a sample plot of SDI *versus* run IDs using a subset of the data presented in Fig. 7a. The advantage of SDI is that it is a universal platform and a standardized methodology for comparison of trends in the same graph irrespective of the mean, the nominal value, assay type, or assay performance. Furthermore, SDI adds granularity to individual QC performance. The capability to have a view of cumulative QC data in a single plot is a useful tool in identifying systematic errors that could impact all QC levels or those which only affect a subset.

For QC47/12, the pre-established QC mean and the SD were 32.3 IU/L and 4.8 IU/L, respectively. One of the shifted samples was measured at 24.2 IU/L. The SDI for this shifted sample is:

$$ SDI=\frac{24.2-32.3}{4.8}=-1.69 $$

The target SDI is 0.0 which would demonstrate that the performance of an individual run is the same as that of the group (of 30 or more runs). SDI of ± 1.0 is considered acceptable although an indication that the assay must be closely monitored. SDI levels between ± 1.0 and 1.5 point to an issue with the assay and call for an investigation. SDI levels ≥ ± 2.0 are considered unacceptable; at these levels, the laboratory should stop testing, troubleshoot, and improve the assay performance before resumption of testing.

## PREVENTION OF ASSAY DRIFT

The performance of LBAs is dependent upon the performance of their constituent biological reagents. These assays heavily rely on protein-protein interactions and the binding properties of assay reagents all of which influence the reactivity of assay components with the target analyte. Over time, these factors render LBAs susceptible to calibration drift. For example, a change to protein deamidation (28) or glycosylation (29) pattern by only one sugar moiety may result in drift. Early signs of calibration drift include but are not limited to changes to the slope and asymptotes of the curve, shift in the assay upper and lower limits of quantitation all of which may result in under- or over-reported sample concentrations. Ultimately, calibration drift leads to misrepresentation of the drug pharmacokinetics. A list of common causes of calibration drift in LBAs are provided as part of the Supplementary Materials.

The following section offers assessment and mitigation strategies for the prevention of assay drift. Irrespective of the root cause, parameters below aid in identifying the performance drift:

### Calibration Curve

- Monitor the concentration-response relationship to ensure that calibrator responses and, in particular that of the zero and high calibrators, have not changed from those observed during pre-study validation. This ensures that LLOQ and ULOQ of the assay are primarily intact and their variability consistent with the that observed in validation (1,2,4).
- Ensure that curve slope and asymptotes are consistent with the variability observed during validation (4).
- Select replacement calibrator stocks/reference standards with performance characteristics most similar to the existing calibrators.
- Ensure previously established equivalence criteria are met.
- Additional recommendations regarding performance of calibration curves can be found in DeSilva *et al.*, Viswanathan *et al.*, and Azadeh *et al.* (1,2,4).

### Quality Controls

- Cross evaluate existing and replacement QCs and/or existing and replacement calibration curves as per previously established procedure. Include negative controls as well as positive controls that span the range of the assay (1–3).
- Track % difference between the measured values of replacement and legacy lots of QCs (refer to sections on Qualification against an Existing Qualified Lot and Qualification in the Absence of an Existing Qualified Lot).
- Long-term monitoring should include trending of the above-mentioned % difference to the legacy lot (or the nominal value if a legacy lot is unavailable) (section on QC Performance Trending).
- Inclusion of dilutional QC is recommended (3).
- Ensure previously established equivalence criteria are met.

### Gold Standard Samples (Proficiency Panel)

Gold standards such as USP or WHO standards may only be applicable to clinical laboratory testing, but when available:

- Evaluation of the gold standard samples along with the assay QCs aides in the identification of the calibration curve drifts as well as in the qualification of the replacement QCs (4).
- If gold standard samples do not exist, a panel of study samples with adequate stability may be reserved and used as gold standard in future replacement lot qualifications (26).

Although control charts are effective monitoring tools, they only utilize measured concentrations of the QCs which are derived from their respective calibration curves. This means that both the calibration curve and the QC lot are made of the same reagents. Cross evaluation of existing and replacement is a critical approach to proper trending and to preventing drift. In this regard, it is important to retain legacy QC lots and bridge them to the newer batches.

The most effective method for trending and for monitoring drift is cross evaluation where any given set of QCs is evaluated against both existing and replacement calibration curves to assess their performance. Other cross evaluation methods which involve assessing existing and replacement QCs against one calibration curve are also helpful although not as informative as the first methodology.

The following are key in not only reliable trending but also in detection of drift

1. A properly qualified matrix pool—the validated test method should clearly define assessment design and acceptance criteria for the qualification of a replacement matrix pool (Qualified Matrix Pool section).
2. Appropriate number of runs—helpful in minimizing variability (1, 2, and Qualification of QCs section).
3. Adequate/appropriate number QC sets per run—most agency guidance as well as a multitude of lead publications have offered recommendations (1–3).
4. Appropriate positional variability—place QCs in the upper left and lower right quartiles of the microtiter plate or adequately and appropriately in the beginning and end of the run (Using Lever-Jennings Principles to Trend QC Performance in Ligand Binding Assays).
5. Introduction of conditions that bring about variability early in method development and during pre-study validation: different analysts, different days, multiple mini-scale preparations all intended to capture variability factors.

## DISCUSSION

Successful management of ligand binding assay life cycle is demonstrated through achievement of consistency in the performance of assay quality controls. As QCs are critical monitoring tools, it is important that laboratories establish standard procedures for their preparation, qualification, and performance trending. This publication has aimed to provide guidelines and best practices pertaining to LBA QCs on subject matters not addressed by regulatory agencies and at the same time, to establish consensus within the bioanalytical community. The authors have presented methodologies for the qualification of replacement QC lots as well as offered practical approaches to trending LBA QC performance. This paper has additionally addressed a variety of questions regarding handling and management of QCs and serves as a reference document for bioanalytical laboratories.

## Electronic Supplementary Material

ESM 1 (DOCX 13 kb)
