# Supplementary material for: Quality Controls in Ligand Binding Assays: Recommendations and Best Practices for Preparation, Qualification, Maintenance of Lot to Lot Consistency, and Prevention of Assay Drift
Source: AAPS J. 2019 Jul 11;21(5):89. doi: 10.1208/s12248-019-0354-6 (PMC6647453; doi:10.1208/s12248-019-0354-6)
Supplement: Supplementary file 1 — (DOCX 13 kb) [file 12248_2019_354_MOESM1_ESM.docx]

| **Source** | **Mechanism** |
| --- | --- |
| Reference Standard | - Unintended change in post-translational modifications - Unintended change in manufacturing or bottling process |
| Matrix Pool | - New pool may consist of a more or less heterogeneous population and change the matrix interference pattern - Components of the pool were processed differently |
| Critical Reagents | Changes to the purity, binding affinity, specificity of capture or detection antibodies/agents and consistency in labeling efficacy of key reagents |
| Noncritical Reagents | Change in the lot, purity, or specificity of secondary detection agents and other assay components |

Table IV. Potential Sources of Calibration Drift in Ligand Binding Assay
